# High-Performance Zeolite Membranes and Natural Gas Upgrading

**DOI:** 10.3390/membranes15120372

**Published:** 2025-12-03

**Authors:** Margarita Kuznetsova, Christophe Castel, Bernardetta Addis, Veronica Piccialli, Eric Favre

**Affiliations:** 1 Université de Lorraine, CNRS, LRGP, F-54000 Nancy, France; margarita.kuznetsova@univ-lorraine.fr (M.K.);; 2 Université de Lorraine, CNRS, LORIA, F-54000 Nancy, France; 3 Dipartimento di Ingegneria Civile e Ingegneria Informatica, Università degli Studi di Roma Tor Vergata, Viale del Politecnico 1, 00133 Rome, Italy

**Keywords:** natural gas, membrane separation, zeolites, process optimization, concentration polarization, technic-economical analysis

## Abstract

Natural gas is currently increasingly used in an energy transition framework and systematically requires upgrading processes in order to respect pipeline specifications. Carbon dioxide, and in some case hydrogen sulfide removal, is the major target of the purification step and can be achieved thanks to gas liquid absorption with chemical solvents or membrane separation. A systematic comparison of the cheap, currently used polymeric membranes and an expensive, high-performance zeolite material is reported on a natural gas upgrading case study (CH_4_/CO_2_ mixture), thanks to a dedicated process synthesis and optimization code (MIND). The zeolite membrane is shown to offer a simple, cost-effective one-stage process, while polymeric materials require more expensive classical two-stage processes. In a second step the impact of concentration polarization is more specifically investigated, through a process simulation study. The zeolite membrane remains the simplest, best cost-effective and most interesting process (one stage without compression, expander or vacuum pump).

## 1. Introduction

Natural gas (NG) is a key fossil energy resource, considered as the most attractive fuel for power generation in the transition to renewables in the next decades [1]. It corresponds to around 24% of the world’s primary resources of energy with an increasing demand of 40% [2]. NG from hydrocarbon fields is classically transported via pipelines. Two major processes are then required for that purpose in order to meet transport specifications: dehydration and sweetening, with a final target methane purity of 98% [3]. CO_2_ removal is by far the major objective of the sweetening/upgrading step, given the quasi-systematic occurrence of CO_2_ with CH_4_ in gas fields, with contents ranging from less than 1% to 70% [4]. Other compounds such as H_2_S, hydrocarbons (C_2_ to C_5_), nitrogen or helium can also be incidentally found. The CO_2_ removal operation is the priority because it enhances the energy content, decreases the volume of gas to be transported and reduces pipeline corrosion. Over decades of research efforts on NG sweetening, different processes such as cryogeny, adsorption, absorption and membranes have been investigated, and applications have been found, amine absorption as the most developed commercial technology [5]. Nevertheless, the high energy requirement, solvent degradation and equipment corrosion issues of the amine units have stimulated the search for alternative upgrading technologies. To that respect, membrane separation is showing an increasing interest, showing the largest growth rate for NG upgrading today [6].

Polymeric materials are mainly used today for membrane gas separations, with two families for natural gas sweetening at industrial scale: cellulose acetate (CA) and polyimides (PIs) [3]. In order to limit methane losses, a two-stage process design, with a permeate-to-feed recycling loop is most often applied [7]. The need for increased CO_2_/CH_4_ selectivity has been recognized for decades in order to improve the energy and cost efficiency of membrane units [8]. Early molecular simulations have suggested that zeolites can show very high CO_2_/CH_4_ selectivities [9]. A large number of publications have indeed reported impressive permeances and selectivities with chabazite, including Si-CHA [10], SAPO-34 [11,12,13], SSZ-13 [14,15] or DDR zeolite materials [16,17]. Alternatively, several families of high-performance inorganic membrane materials, such as silica, Carbon Molecular Sieves (CMSs) and Metal Oxide Frameworks (MOFs), have been reported to show breakthrough improvements compared to existing NG purification polymeric membranes. A major issue for decades has been the production of large-scale zeolite membranes and modules; however, suppliers of zeolite membrane modules do exist today [17,18,19].

This situation logically raises the key question of the impact of high-performance membrane materials for NG upgrading. A close combination of materials and process design research is required in order to address this question [2]. A Process Systems Engineering (PSE) strategy is best suited in order to offer a systematic analysis of the potential of high-performance materials for NG sweetening, with a cost function as the ultimate objective. A limited number of studies recently explored the interest of carbon and CMS membranes for biogas and NG treatment [20,21].

The objective of this study is to evaluate the impact of a high-permeance/high-selectivity zeolite material, compared to currently used polymers (cellulose acetate and polyimides) for an NG upgrading case study. A rigorous tailor-made process synthesis program, making use of advanced superstructure optimisation computing methods, MIND, is used for that purpose [22]. The objective is to evaluate the impact of high-performance membranes in terms of process design, energy efficiency, surface and cost for NG treatment. The influence of concentration polarization effect is also presented.

## 2. Process Design Methodology

### 2.1. Membranes for CO_2_/CH_4_ Separation

**Figure 1:**
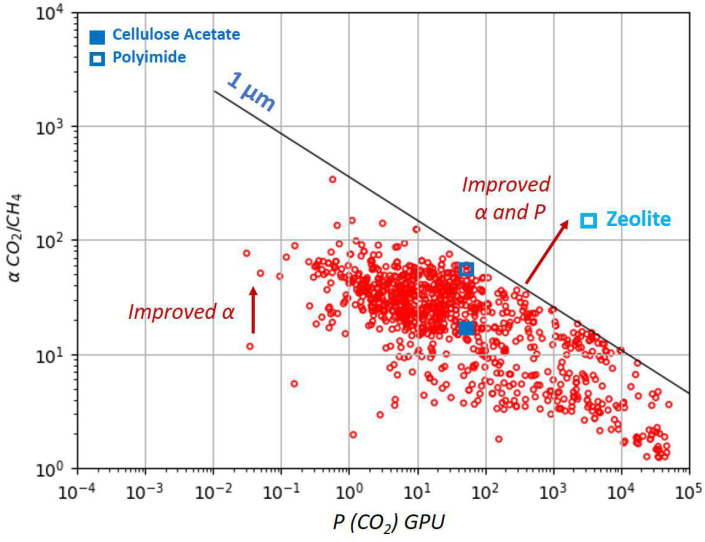
Trade-off curve for the CO_2_/CH_4_ gas pair based on permeability for different polymeric materials. The selectivity–permeance trade-off is shown assuming a skin-layer thickness of 1 µm (1 Barrer = 1 GPU), together with the performances of two polymeric membranes (polyimide and cellulose acetate) and one inorganic zeolite membrane. The figure is adapted from references [23,25].

The well-known permeability–selectivity trade-off for polymeric materials is illustrated by the Robeson plot in Figure 1 [23]. Among various materials synthetized and studied in the laboratory, only two glassy polymers are widely commercially employed for ${\mathrm{CO}}_{2}$ removal from NG—cellulose acetate and polyimide. They typically exhibit ${\mathrm{CO}}_{2}$/${\mathrm{CH}}_{4}$ selectivity in the range of 15–40 [24]. The upper-bound curve of the Robeson plot shows a current theoretical maximum of the polymeric membrane performances. However, materials such as zeolites surpass the upper bound showing simultaneously higher selectivities and permeabilities than conventional membranes, being placed in the upper right corner as it is shown in Figure 1 [25]. A thickness of a selective membrane layer of 1 µm is taken for the trade-off permeance calculation. Characteristic parameters of various zeolite membranes for CO_2_/CH_4_ separation are given in Table 1 [26]. Robeson plots for more zeolite structures are reviewed elsewhere [27].

Zeolites are advantageous because they generally possess greater chemical and thermal stability compared to conventional polymers. Moreover, zeolites can operate at harsh conditions [28]. They do not undergo swelling or plasticization, as well as have regular pore structure of molecular dimensions, allowing separation due to molecular sieving effect [29]. However, combination of high selectivity and permeability gives rise to concentration polarization (CP), reducing the separation performance of zeolite membranes. In gas separation, CP effect becomes significant with permeance of more than 1000 GPU (Gas Permeation Unit, 1 GPU = $3.348\;\times \;10^{-10}\mathrm{mol}m^{-2}s\mathrm{Pa}$) for the fastest compound, while selectivity is more than 100 [30]. It becomes significant when operating at high pressures [31]. The importance of accounting for CP in process design has been highlighted [32]. Therefore, the effect of CP in zeolite membranes is discussed, as well as its influence on process design of CO_2_ removal from natural gas.

**Table 1:** Selectivity and permeability of zeolite membranes for CO_2_/CH_4_ separation. This table is adapted from reference [26].

Material	CO_2_/CH_4_ Selectivity (22–35 °C)	CO_2_ Permeability (Barrer)	References
DD3R	500	687	[33]
DD3R	500	896	[34]
T	400	2746	[35]
AlPO-18	220	15,522	[36]
SSZ-13	406	5970	[37]
SAPO-34	170	1791	[12]

### 2.2. Optimization Method for Membrane Gas Separation

In this study, the optimization method aims to find optimal membrane configurations for the removal of CO_2_ from the natural gas (binary CO_2_/CH_4_ mixture). The optimal process configurations presented in this paper have been obtained using a dedicated computational tool (MIND software version 1) specifically developed for membrane gas separation systems. Based on predefined separation specifications (feed composition, and permeate and/or retentate composition), the program determines the optimal process flowsheet and associated operating conditions through a global optimization algorithm employing an objective cost function. The tool relies on a rigorous mathematical framework for membrane module simulation, which can be extended to multicomponent mixtures [38]. Feasible interconnection schemes are systematically explored for configurations comprising one, two, or three stages, including both interstage and self-recycling loops. A distinctive feature of this program, compared with most methodologies available in the literature, lies in its capability to accommodate variable pressure ratios within each stage, with vacuum operation considered as a viable option. The range of admissible operating conditions (pressures, areas ...) is constrained by technological limitations. Compressors and vacuum pumps are incorporated into recycling loops whenever pressure variations occur across the interconnections. The global cost function described in Table 2, adopted as the objective function, integrates both capital expenditures (CAPEX)—including compressors, vacuum pumps, and membrane modules—and operating expenditures (OPEX), encompassing energy consumption, membrane replacement, and operation and maintenance costs. The comprehensive mathematical formulation and the corresponding optimization strategy are detailed in [22,25,39]. The optimization algorithm yields physically meaningful and technically feasible configurations combined with the minimal total separation cost, as the objective function. The cost functions used for the technic-economical analysis taken from Ramirez-Santos et al. [39] are listed in Table 2.

### 2.3. Natural Gas Sweetening: Case Study

**Figure 2:**
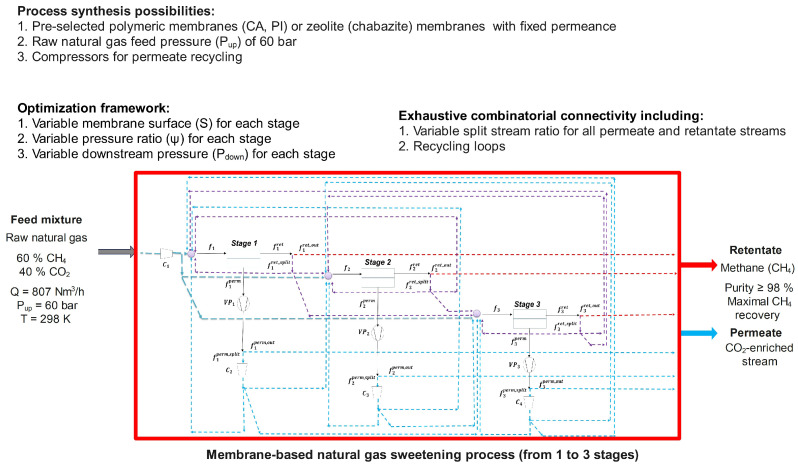
Overall process synthesis framework applied in this study. A membrane separation process including up to 3 stages with compressors is used. Multiple connection possibilities including recycling loops is applied to obtain pure methane. The different configuration possibilities and operating variables are taken into account in order to achieve the lowest production cost (i.e., objective function, detailed in corresponding Table 2 and Table 3).

This case study considers a natural gas stream with a composition of 60/40 mol.% in CH_4_/CO_2_ and an inlet flowrate of 10 mol/s (807 N$m^{3}h^{-1}$). It was assumed that the feed gas mixture has been previously dehydrated to remove any water vapour, which is a common operation in natural gas upgrading [6]. In addition, heavier hydrocarbons (C_2_+) were presumed to be removed during the pre-treatment step. Therefore, the separation of a CH_4_/CO_2_ binary mixture was considered under an idealized dry-gas scenario. The studied feed composition corresponds to the natural gas reserves with low methane content [4]. The raw natural gas was naturally recovered from the sources at high pressure of 20–80 bar [40]. Therefore, the inlet pressure of 60 bar was considered for simulations with temperature of 298.15 K. The main goal is to recover CH_4_ at the retentate with high purity exceeding 98% to meet the imposed regulatory requirements, with minimization of CH_4_ losses as the optimization constraint (Figure 2). Process configuration includes from one to three membrane stages for each membrane material. The pressure ratio (Ψ) of each module varies in terms of downstream pressure allowed for optimization, while the upstream pressure was fixed and identical across all stages and configurations. The operating temperature was fixed and not included as an optimization variable. Isothermal conditions were assumed for all membrane stages [41,42], and Joule–Thompson effects due to gas expansion were not considered. In the present multi-objective optimization, temperature was treated as a secondary-level variable and could be incorporated as an additional parameter in future work.

The main objective of this optimization case is to compare high-performant zeolite membranes with conventionally used polymers by identifying the most efficient process configurations, with fixed operating conditions by minimizing the cost. The final product, methane (CH_4_), must meet the required specifications in terms of purity and maximum recovery, with respect to imposed optimization constraints. Therefore, CO_2_-selective membranes, polymers (CA and PI) and high-performant zeolite membrane (chabazite) are considered for the process synthesis, and their characteristics are listed in Table 4. Polymeric membranes are known for their low production cost, while zeolites are significantly more expensive and have larger capital cost [43]. In our process optimization, a cost of EUR 50/m^2^ was considered for polymers, while the cost of inorganic membranes typically varies from EUR 1000 [44] to EUR 5000/m^2^ [45]. In this study, an estimated cost of EUR 2000/m^2^ was used for the zeolite membrane. The trade-off between high performance and associated high cost are therefore discussed.

In the process optimization, recycling loops were allowed for both the retentate and permeate streams, with compressors used to efficiently recycle the downstream flows. The overall cost function (SC_CH_4__) was minimized while targeting a methane purity above exceeding 98% and maximal recovery rate. A set of optimal configurations was generated by the simulator.

**Table 4:** Membrane characteristics used for the process synthesis in the current study.

Material	P_CO_2__ (GPU)	P_CH_4__ (GPU)	Cost (EUR/m^2^)	References	Comments
Cellulose acetate (CA)	60	3	50	[46]	First commercialised membrane material for CO_2_/CH_4_ separation
Polyimide (PI)	60	1	50	[46]	Second-generation polymeric membrane material (improved selectivity, close to trade-off limit)
Zeolite	3500	22	2000	[47]	High-performance inorganic membrane material (breakthrough permeance and selectivity)

### 2.4. Concentration Polarization: Post-Treatment Calculations

During the optimization procedure, the concentration polarization phenomena was not directly accounted for membranes during the process architecture optimization. This is related to the high complexity of integration of the CP in the process synthesis. Its influence is rigorously studied for the zeolite membrane module during subsequent computer simulations with considering different module geometries. An in-house software based on computational fluid dynamics (CFD) approach has been previously developed and reported by Abdul Majid et al. [48,49] to predict membrane separation characteristics considering local concentration polarization along a membrane fiber. The model and correlations are valid under laminar flow conditions, and concentration polarization is considered in the retentate side. This requires an estimation of the mass transfer coefficient $k_{ret}$, which is strongly related to the hydrodynamics in membrane modules. It is classically estimated through dedicated correlations between Sherwood, Schmidt and Reynolds dimensionless numbers. A brief description is provided in Appendix A.

## 3. Results and Discussion

### 3.1. Process Configurations for Natural Gas Purification

**Figure 3:**
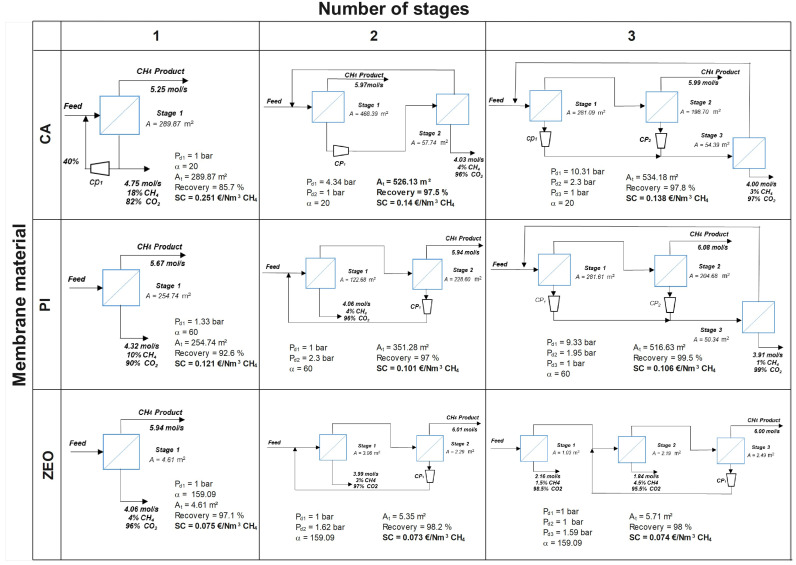
The best process configurations obtained with cellulose acetate (CA), polyimide (PI), and zeolite (ZEO) membranes for up to three stages.

Figure 3 shows the optimal solution for process configuration for CO_2_ removal from NG with up to three membrane stages. Commercially available polymeric membranes, as well as high-performant zeolites have been considered and compared. Note that target CH_4_ purity in the retentate of 98% with minimal methane loses are the optimization constraints.

As can be seen, for classical polymers, the desired methane purity can be theoretically achieved with a single-stage permeation unit. However, CH_4_ losses are very high, achieving 7–14%, as methane also passes through the membrane. In the literature, losses of 12% for a single stage have been reported by Scholes et al. [4]. For first-generation CA membrane (α=20), a significant recycling part of the permeate is required. Adding a compressor on the downstream side drastically increases the overall process cost. A more selective PI membrane (α=60) can operate without recycling loops, which reduces the total process cost, but the methane recovery remains poor. Interestingly, with zeolite membranes, CH_4_ conforming to the pipeline specifications with recovery of 97% can be theoretically obtained in only a one-stage process without any stream recycling. Higher selectivity (α=159.09) allows the production of a gas with higher purity. Moreover, the very high CO_2_ permeance results in less membrane area needed for NG upgrading: the separation is feasible with only 4.61 m^2^ of zeolite. A factor of around 60 is thus gained in surface area compared to conventional polymers. In general, one stage configuration is simple to handle, has a low footprint and has no rotation parts which are easy in maintenance, which is associated with high methane losses [7].

The advantages of zeolite single-stage configurations are as follows: lower membrane area needed for the separation, lower associated membrane cost, and lower energy consumption as no recycling is needed. As a result, operation of single-stage zeolites is technically and economically interesting, reducing the cost of the membrane unit and simultaneously maximizing methane recovery. Nevertheless, remember that this configuration is generated by a mathematical optimization algorithm where concentration polarization is not taken into account. For rigorous process design, the influence of CP effect on separation performance depending on module geometry is discussed later.

Two-stage cascade configurations with polymeric membranes are typically used in industry for natural gas upgrading. In our analysis, the optimal process solutions include both so-called “fast” and “slow” configurations, depending on the membrane material (Figure 3). For the CA membrane, a fast-compound configuration is obtained: purified CH_4_ is recovered in the retentate of the first stage, while the permeate is compressed and further treated in a second stage; the retentate from the second stage is recycled to the feed of the first stage. By contrast, for the PI and zeolite membranes, the best solution is a slow configuration. Here, the final product is recovered in the retentate on the second stage, while the permeate of this second stage is recompressed and recycled to the feed to improve methane recovery. The retentate from the first stage is directly fed to the second membrane module, and the collected permeate is enriched in CO_2_. It is important to mention that both process designs, identified through process synthesis for two-stage configurations, are consistent with established industrial practice for natural gas upgrading. During the optimization, no partial recycling loops were proposed by the algorithm.

As shown, in the two-stage configuration with the CA membrane, a feasible solution is obtained that satisfied the imposed optimization constraints (methane purity and recovery). Purified CH_4_ with the target purity of 98% is recovered in the retentate on the first stage. The permeate from the first stage is treated in the second module to reduce methane losses. A CH_4_ recovery of 97.5% is achieved with a total membrane area of 526.13 m^2^, consisting of 468.39 m^2^ for the first module, and 57.74 m^2^ for the second. It worth noting that, in multi-stage membrane systems (both with two- and three- stage configurations), the membrane surface area of each stage results directly from the optimization. As shown in Table 2, the membrane area ($A_{ms}$) is directly related to the CAPEX through the corresponding equations. This cost-based optimization provides an effective way to deal with the compromise between the membrane CAPEX, which is directly related to the membrane area, and the compressor cost (CAPEX and OPEX).

For PI and zeolite membranes, the best solution is the slow-compound configuration, where retentate from the first stage is directly fed into the second membrane module, and the final product is recovered in the retentate of the second stage. The permeate of the second stage is recompressed and recycled to improve methane recovery. PI membranes achieve a CH_4_ recovery of 97%, whereas zeolites reach an excellent 98%. In this mode, the purification cost is more attractive: due to higher membrane selectivity, less area is needed, and a smaller volume of gas is recompressed. For the PI, the total membrane area is 351.28 m^2^ (122.68 m^2^ for the first stage and 228.60 m^2^ for the second), whereas, for the highly efficient zeolites, only 5.35 m^2^ of total membrane area is needed. Moreover, the techno-economic analysis shows that operating zeolite membranes is approximately twice as cost-effective as using CA membranes: EUR 0.073/Nm^3^ CH_4_ for zeolites compared to EUR 0.14/Nm^3^ CH_4_ for CA. Therefore, among the two-stage configurations, zeolite membranes operating in the slow configuration represent the most economically attractive option while offering advantageous operability. The capital and operating cost are higher for the two-stage configuration compared to the single-stage one due to two modules and recompression [40]. However, methane losses are minimized, and it can be efficiently recovered. Our process design simulations serve as a decision tool to make the best choice depending on the operating conditions and desired outcome.

At first glance, the three-stage configuration might seem more interesting; however, this is not the case. The two-staged slow-compound scheme proves to be more advantageous, offering almost the same separation efficiency with similar cost, but with reduced installation complexity and simpler operation. For zeolite membranes in particular, the differences between the two configurations are minimal. As observed, no expander or vacuum pump is included in the final configurations. However, these components were considered in the MIND framework with their corresponding cost functions (Table 2 and Table 3). The optimization results show that the solutions including these elements are not beneficial from the technic-economical point of view.

**Figure 4:**
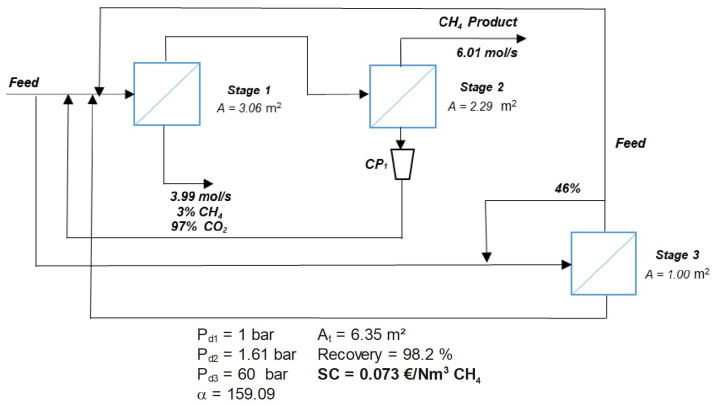
Example of a configuration obtained with MIND for zeolite membrane under imposed three-stage separation process. The solution includes partial recycling loops, and a minimal membrane area of 1 m^2^ is respected for the third stage. Although this configuration has no engineering relevance, it highlights that the target separation can be achieved efficiently with only two stages.

Interestingly, one of the optimization results for zeolite membranes includes a configuration with internal loops having no clear impact on process performance. This peculiar solution, depicted in Figure 4, appears to satisfy the imposed three-stage optimization constraint, with the third stage having a minimal membrane area of just 1 m^2^. This underlines the strong capability of MIND to find non-standard solutions, including partial recycling loops, which satisfy the imposed constraints. However, this configuration is considered a forced solution and, from an engineering point of view, has no practical interest. It serves as further proof that efficient removal of CO_2_ from raw natural gas can be achieved with zeolite membranes in two stages at minimal cost.

### 3.2. Effect of Concentration Polarization

As seen, theoretically CO_2_/CH_4_ separation is feasible in one stage using high-performant zeolite membranes with the optimal membrane surface of 4.61 m^2^ for our case study. The aim of further computer simulations is to evaluate the effect of concentration polarization on the process feasibility, productivity, separation performance, and economical interest. Within the scope of this study, we focus on the single-stage configuration to perform numerical simulations.

As there is no (or very few) industrial scale manufactures of zeolite membranes for gas separation, we take three different module geometries reported in the literature, notably, hollow fiber bundle [16], monolith [19], and tubular [47,50] membranes. The gas mixture is fed from the lumen side of a fiber membrane with “inside-out” feed. Geometric parameters are listed in Table 5. As observed, zeolite membranes have rather large diameters compared to the conventional hollow fiber polymers, which is explained by the differences in manufacturing procedures [48].

#### 3.2.1. Effect of Geometry

A single-stage configuration was considered with a fixed membrane area of 4.61 m^2^, with numerous parallel fibers uniformly fed, and three different inner diameters listed in Table 5. The operating conditions were kept identical to those used in the case without CP. Since the total membrane area was fixed, the number of fibers varied with the inner diameter, resulting in different gas velocities inside the fibers. Nevertheless, the flow regime was maintained within the laminar regime (Re < 2000). Table 6 reports the effect of fiber diameter on the CH_4_ composition on the retentate and on CH_4_ recovery, subject to the initial optimization constraints. Note that, in the absence of concentration polarization (CP), the retentate methane purity is 98%, and the recovery is 97.1%. As observed, when CP is accounted for, the CH_4_ purity decreases to 76.2% for the $d_{int}$ of 7 mm, to 85.0% for $d_{int}$ of 2.4 mm, and to 92.6% for the smallest $d_{int}$ of 0.9 mm. The observation that the recovery rate remains almost the same for four different cases may seem surprising. This behaviour results from a compensation phenomenon: while CH_4_ purity decreases in the retentate, the retentate flowrate increases as the permeate flowrate decreases due to CP. However, this trend is not a general rule but a particular solution for this set of constraints, feed composition, as well as permeance values.

As seen, the CP effect is more pronounced for larger fiber diameters and less significant for smaller ones. Therefore, effective separation with the same membrane surface of 4.61 m^2^ and operating pressures is not achievable when CP is taken into account for zeolite membranes. Indeed, the driving force for permeation is altered by the accumulation of less permeable methane species in the boundary layer, leading to less effective separation. However, hollow fibers with diameters of approximately 0.9 mm appear particularly promising, as the concentration polarization effect is less pronounced. This can be attributed to their higher surface-to-volume ratio and improved convective mass transfer [51].

The effect of fiber length on concentration polarization, and consequently on overall separation performance, may seem counter-intuitive. If the inner diameter of fibers and operating conditions are kept constant (temperature, pressure), the most relevant parameter in not the fiber length *L* but the ratio between the initial velocity *u* and the length, u/L, which follows from the equations in Appendix A. In shorter fibers, velocity *u* is smaller, while, in longer fibers, velocity *u* is bigger, but the u/L ratio is the same for the same simulation conditions. Therefore, from purely concentration polarization point of view (neglecting the pressure drop, for example and other effects), the membrane area is the most important, and the fiber length itself does not impact the outcome.

#### 3.2.2. Sensitivity Analysis

**Figure 5:**
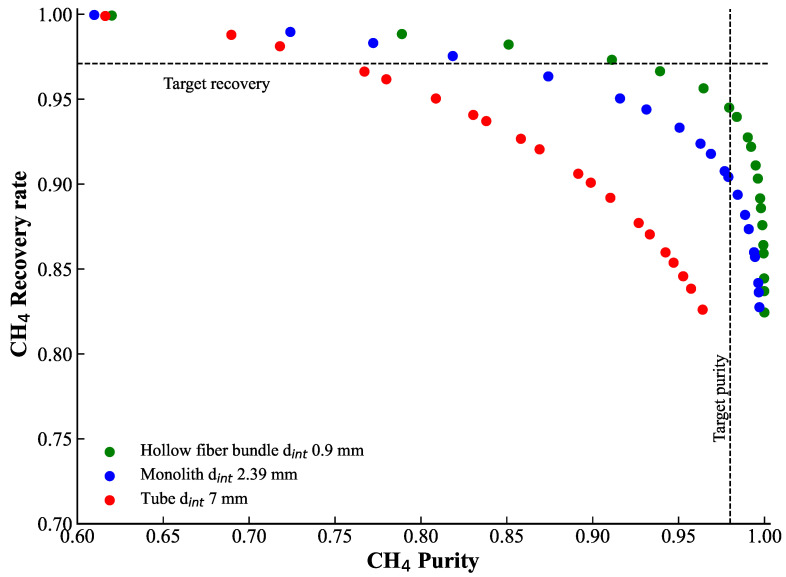
Results of the sensitivity analysis obtained by varying the membrane area from 4.61 m^2^ to 25 m^2^ for the three fiber diameters (0.9, 2.4, and 7 mm). The intersection of the dotted lines represents the reference case without considering concentration polarization.

Since concentration polarization impacts the overall separation performance by decreasing the methane content in the retentate, a sensitivity analysis was provided to evaluate the feasibility of a single-stage separation with zeolite membranes considering CP. The membrane area was varied from 4.61 m^2^ to 25 m^2^ for all three fiber diameters. The results are presented in Figure 5. The black dotted lines correspond to the target performance obtained from process optimization (CH_4_ purity of 98% and CH_4_ recovery of 97%). As observed, this performance cannot be achieved under CP conditions. However, thin hollow fibers exhibit promising results, reaching CH_4_ purity of 98% and a recovery rate of about 95% with the membrane area close to 8 m^2^. This is considered the best-case scenario with CP.

As a result, CP may strongly affect the performance of membrane-based separation processes. It is therefore important to account for membrane geometry and hydrodynamic conditions; otherwise, the expected performance may be overestimated. For example, for three different module geometries with lumen-side feed, the initial characteristics obtained without considering CP cannot be recovered simply by increasing the membrane surface area.

### 3.3. Synopsis

A synopsis of the process configurations obtained from the optimization synthesis, followed by concentration polarization (CP) numerical simulations and sensitivity analysis, is presented below. As depicted in Figure 3, for polymeric membranes, the optimal two-stages solutions provided the best compromise between cost, complexity, and efficiency. These configurations included permeate recycle for cellulose acetate (CA) membranes and retentate recycle for polyimide (PI) membranes. Interestingly, for zeolite membranes, the best configuration corresponded to single-stage separation module of 4.61 m^2^. Note that optimization constraints were CH_4_ purity above 98% and maximum recovery.

**Figure 6:**
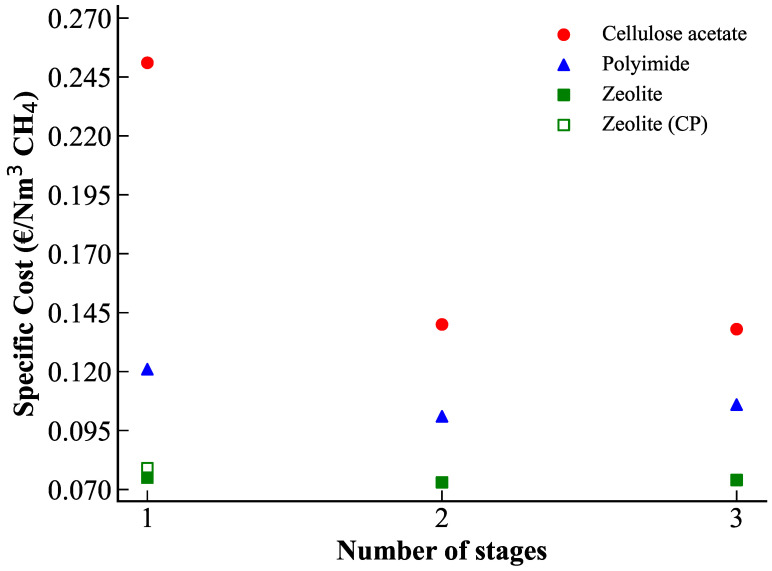
Specific cost of CH_4_ purification for the optimal configurations obtained with different membrane types as a function of the number of stages. For zeolite membranes, both the cost without concentration polarization and the cost including CP (best-case scenario) are shown.

However, concentration polarization (CP) effects can become severe for highly efficient zeolites. Post-treatment CP simulations showed that the target methane purity and recovery rate can not be achieved when CP is accounted for, considering existing membrane modules reported in the literature (lumen-side feed hollow fibers, monoliths, tubes). This analysis highlights the importance of improving the shaping and geometrical design of zeolite membranes to reduce CP impact. Even increasing the total membrane surface area does not fully restore the performance predicted without CP. The best-case scenario was obtained for hollow fibers with an internal diameter of 0.9 mm and a total membrane area of approximately 8 m^2^. Thinner fibers experience weaker CP effects, whereas tubular geometries with diameters of 7 mm exhibit strong polarization. A techno-economic analysis, summarized in Figure 6, confirms that, although zeolite membranes are more expensive in terms of material cost, they offer significantly lower operational expenses. Overall, zeolite membranes demonstrate highly competitive specific separation costs, even when CP effects are considered.

## 4. Conclusions

This study intended to rigorously evaluate the interest of high-performance inorganic membranes (zeolite) compared to the currently used first (CA) and second generation (PI) polymeric materials for natural gas upgrading. The high performances of zeolites, both in terms of permeance and selectivity, indeed offer attracting perspectives, at the expense, however, of a very high cost. A systematic process synthesis study, thanks to a tailor made optimization code (MIND), has been performed in order to identify the best cost-effective process designs with one, two or three stages for the three types of materials.

Zeolite membranes show the unique opportunity to achieve the target natural gas purification performances with one single stage showing the simplest design (no compression, no expander, no vacuum pump) at the lowest cost. The high performances thus compensate the high cost: a very low membrane surface area is needed thanks to the high permeance, and a high process selectivity is obtained thanks to the high material selectivity. Polymeric membranes require two-stage processes with a high overall cost. In that case, the optimal process designs generated by the optimization code are consistent with existing designs used for natural gas upgrading, showing that relevant process solutions are obtained by process synthesis.

In the second step, the impact of concentration polarization on the optimal zeolite design has been investigated with a tailor-made simulation code. Three different geometries have been compared, showing different separation performances. A minimal membrane diameter is needed in order to minimize concentration polarization. This result suggests dedicated efforts in order to promote production technologies leading to small-diameter inorganic membranes. Alternatively, turbulence promoters could be proposed for large-diameter tubular membranes. All in all, the zeolite membrane remains the most interesting solution, even when the concentration polarization penalty is included.

The pragmatic approach has been proposed here: process optimization without concentration polarization first, and dedicated process design with a simulation code taking into account concentration polarization in a second step. The combined process synthesis/concentration polarization optimization is clearly a perspective for future work. This requires, however, significant efforts, both in terms of methodology and computing effort: concentration polarization necessitates geometry and operating conditions to be defined (diameter, length, velocity), leading to a very large increase of the number of variables to be explored in the optimization space, with a complex combinatorial interplay. To our knowledge, no study has addressed this challenge up to now.

Finally, the simultaneous natural gas purification (retentate purity) and carbon capture (permeate purity) target, a major challenge of sustainable energy transition, could also be explored, ideally with a single-stage design, given the separation perspectives offered by zeolite and high-performance membrane materials. In our case, one strategy to push methane recovery beyond purification guidelines would be to apply a lost product price in the set of data. Moreover, the high CO_2_ permeate purity that would result is likely to be of interest for (Carbon Capture and Use) or EOR (Enhanced Oil Recovery).

## Figures and Tables

**Table 2:** Cost equations used in this study for technic-economical analysis to determine product separation cost.

Equipment cost		
$I_{m_{s}}=A_{m_{s}}\;\cdot \;K_{m}$	(1)	Membrane cost
$I_{mf_{s}}={\left(A_{m_{s}}/2000\right)}^{0.7}\;\cdot \;K_{mf}\;\cdot \;{\left(p^{up}/55\right)}^{0.875}$	(2)	Membrane frame cost
$I_{c_{s}}=C_{c}\;\cdot \;{\left(W_{{cp}_{s}}/74.6\right)}^{0.77}\;\cdot \;\left(MPF_{c}+MF_{c}-1\right)\;\cdot \;UF_{1968}\;\cdot \;K_{er}$	(3)	Stage compressor cost
$I_{c_{f}}=C_{c}\;\cdot \;{\left(W_{{cp}_{f}}/74.6\right)}^{0.77}\;\cdot \;\left(MPF_{c}+MF_{c}-1\right)\;\cdot \;UF_{1968}\;\cdot \;K_{er}$	(4)	Feed compressor cost
$I_{c_{prod}}=C_{c}\;\cdot \;{\left(W_{{cp}_{prod}}/74.6\right)}^{0.77}\;\cdot \;\left(MPF_{c}+MF_{c}-1\right)\;\cdot \;UF_{1968}\;\cdot \;K_{er}$	(5)	Retentate compressor cost
$I_{exp}=C_{exp}\;\cdot \;{\left(W_{exp}/0.746\right)}^{0.81}\;\cdot \;\left(MPF_{c}+MF_{c}-1\right)\;\cdot \;UF_{2000}\;\cdot \;K_{er}$	(6)	Expander cost
$I_{vp_{s}}=C_{vp}\;\cdot \;\left(W_{vp_{s}}\right)$	(7)	Vacuum pump cost
**Capital expenditures**		
$PFC=I_{c_{f}}+I_{c_{prod}}or\;I_{exp}+\sum_{s\in S}I_{m_{s}}+I_{mf_{s}}+I_{c_{s}}+I_{vp_{s}}$	(8)	Process facilities capital
BPC=1.12·PFC	(9)	Base plant cost
PC=0.2·BPC	(10)	Contingency cost
TFI=BPC+PC	(11)	Total facility investment
STC=0.10·OPEX	(12)	Start up cost
CAPEX=TFI+STC	(13)	Total capital cost
**Operational expenditures**		
CMC=0.05·TFI	(14)	Contract and material maintenance cost
LTI=0.15·TFI	(15)	Local taxes and insurance
$DL=11\;\cdot \;t_{op}$	(16)	Direct labor
LOC=1.15·DL	(17)	Labor overhead cost
$EC=t_{op}\;\cdot \;W_{tot}\;\cdot \;K_{el}$	(18)	Energy cost
$MRC=\sum_{s\in S}A_{m_{s}}\;\cdot \;\nu \;\cdot \;K_{mr}$	(19)	Membrane replacement cost
OPEX=CMC+LTI+DL+LOC+EC+MRC	(20)	Total operational expenditures
**Annual and specific separation costs**		
$APL=F^{PERM}\;\cdot \;3600\;\cdot \;0.0224\;\cdot \;K_{gp}\frac{X_{CH_{4}}^{PERM}}{X_{CH_{4}}^{RET}}$	(21)	Annual CH_4_ losses
$TAC=CAPEX\;\cdot \;\frac{i\;\cdot \;{\left(1+i\right)}^{z-1}}{{\left(1+i\right)}^{z}-1}+OPEX+APL$	(22)	Total annual costs
$SC_{CH_{4}}=TAC/\left(F^{RET}\;\cdot \;3600\;\cdot \;t_{op}\;\cdot \;0.0224\right)$	(23)	Specific CH_4_ separation cost

**Table 3:** Cost parameters used in Table 2.

Capital cost parameters		
$C_{c}$	1 × 23,000	${\mathrm{USD}}_{1968}$
$C_{vp}$	1000	EUR/kW
$C_{exp}$	420	${\mathrm{USD}}_{2000}$
$K_{m}\left(polymer\right)$	50	$\mathrm{EUR}/m^{2}$
$K_{m}\left(zeolite\right)$	2000	$\mathrm{EUR}/m^{2}$
$K_{mf}$	$2.86\times 10^{5}$	EUR
$K_{er}$	0.9	EUR/USD
$MPF_{c}$	2.9	-
$MF_{c}$	5.11	-
$UF_{2000}$	1.44	-
$UF_{1968}$	4.99	-
**Operational and annual cost parameters**		
ν	0.25	-
$K_{mr}\left(polymer\right)$	25	$\mathrm{EUR}/m^{2}$
$K_{mr}\left(zeolite\right)$	2000	$\mathrm{EUR}/m^{2}$
$t_{op}$	8322	h/year
$K_{el}$	0.08	EUR/kWh
$K_{gp}$	0	$\mathrm{EUR}/{\mathrm{Nm}}^{3}$
i	0.08	-
*z*	15	years
$\eta_{c}$	0.85	-
Φ	0.95	-
γ	1.36	-
*R*	8.314	J/(K·mol)
*T*	293.15	K

**Table 5:** Geometric parameters used to study concentration polarization effect in highly performant zeolite membranes.

N	Inner Diameter, mm	Module Type	References
Case 1	0.9	Hollow fiber	[16]
Case 2	2.4	Monolith	[19]
Case 3	7	Tubular	[47,50]

**Table 6:** Effect of concentration polarization (CP) on the methane mole fraction in the retentate and on the methane recovery for different inner diameters of zeolite fibers (0.9, 2.4, and 7 mm) compared to the reference case without CP. The membrane area is 4.61 m^2^ for all the cases.

Case	CP	Inner Diameter, mm	CH_4_ Purity, %	CH_4_ Recovery Rate, %
Reference	No	—	98.2	97.1
Case 1	Yes	0.9	92.6	97.0
Case 2	Yes	2.4	85.0	97.0
Case 3	Yes	7	76.2	96.9

## Data Availability

The raw data supporting the conclusions of this article will be made available by the authors upon request.

## Nomenclature

Abbreviations	
NG	Natural Gas
CA	Cellulose Acetate
PI	Polyimide
CMSs	Carbon Molecular Sieves
MOF	Metal Organic Framework
PSE	Process Systems Engineering
CP	Concentration Polarization
SC	Specific Cost
CAPEX	Capital Expenditure
OPEX	Operational Expenditure
CFD	Computational Fluid Dynamics
CCU	Carbon Capture and Use
EOR	Enhanced Oil Recovery
GPU	Gas Permeation Unit
Parameters	
$C_{c}$	Compressor Base Cost [USD_1968_]
$C_{vp}$	Vacuum Pump Cost [EUR/kW]
$C_{exp}$	Expander Base Cost [USD_2000_]
$K_{m}$	Unit Cost of Membrane Module [EUR/m^2^]
$K_{mf}$	Base Frame Cost [EUR]
$K_{er}$	Exchange Rate [EUR/USD]
$MPF_{c}$	Material and Pressure Factor for Compressor [-]
$MF_{c}$	Module Factor for Compressor [-]
$UF_{2000}$	Update Factor [-]
$UF_{1968}$	Update Factor [-]
ν	Membrane Annual Replacement Rate [-]
$K_{mr}$	Membrane Replacement Cost [EUR/m^2^]
$t_{op}$	Operation Time per Year [h/year]
$K_{el}$	Electricity Cost Factor [EUR/kWh]
$K_{gp}$	Lost Product Price [EUR/Nm^3^]
*i*	Interest Rate [%]
*z*	Project Lifetime [years]
$\eta_{c}$	Isentropic Compressor Efficiency [-]
Φ	Mechanical Efficiency [-]
γ	Gas Expansion Coefficient [-]
*R*	Ideal Gas Constant [J mol^−1^ K^−1^]
*T*	Temperature [K]
$A_{m}$	Membrane Area [m^2^]

## Appendix A

Governing equations used to account for concentration polarization (CP) during the post-treatment step. A detailed description of the model can be found in Abdul Majid et al. [48].

$$Sh\left(z\right)=\frac{k_{ret}\left(z\right)d}{D_{gas}}\left\{\begin{array}{c} =\left[3.6568+0.2249Gz^{0.4956}exp\left(\frac{-55.9857}{Gz}\right)\right]\ldots \\ {\left[1+98.42\left(1+Gz^{0.24}\right)tanh\left(\frac{0.93}{Sh_{w}}\right)\right]}^{0.03}\;\ldots \\ \ldots {\left[1+0.004\left(1+tanh\left(\frac{61.74}{Sh_{w}}\right)\right)\frac{Gz}{Sc}\right]}^{0.12}if\;Gz\leq 10^{3}\\ =\left(-0.3856+1.022Gz^{0.3366}\right)\ldots \\ {\left[1+98.42\left(1+Gz^{0.24}\right)tanh\left(\frac{0.93}{Sh_{w}}\right)\right]}^{0.03}\;\ldots \\ \ldots {\left[1+0.004\left(1+tanh\left(\frac{61.74}{Sh_{w}}\right)\right)\frac{Gz}{Sc}\right]}^{0.12}if\;Gz\geq 10^{3} \end{array}\right.$$

Therefore,

(A1) $$k_{ret}=\frac{Sh\left(z\right)D_{gas}}{d}$$

(A2) $$\begin{array}{cc} \mathrm{Sherwood}\;\mathrm{number} & Sh_{w}=\frac{max\left({Permeance}_{i}\right)R_{gas}Td}{D_{gas}} \end{array}$$

(A3) $$\begin{array}{cc} \mathrm{Graetz}\;\mathrm{number} & Gz\left(z\right)=\frac{dScRe(z)}{z} \end{array}$$

(A4) $$\begin{array}{cc} \;\mathrm{Schmidt}\;\mathrm{number} & Sc=\frac{µ}{\rho D_{gas}} \end{array}$$

(A5) $$\begin{array}{cc} \;\mathrm{Reynolds}\;\mathrm{number} & Re=\frac{\rho ud_{int}}{µ} \end{array}$$
